# Cost-Effective and Handmade Paper-Based Immunosensing Device for Electrochemical Detection of Influenza Virus

**DOI:** 10.3390/s17112597

**Published:** 2017-11-11

**Authors:** Sivaranjani Devarakonda, Renu Singh, Jyoti Bhardwaj, Jaesung Jang

**Affiliations:** 1 Department of Mechanical Engineering, School of Mechanical, Aerospace and Nuclear Engineering, Ulsan National Institute of Science and Technology (UNIST), Ulsan 44919, Korea; ranjani.devarakonda@gmail.com (S.D.); raina1785@gmail.com (R.S.); 2 Department of Biomedical Engineering, Ulsan National Institute of Science and Technology (UNIST), Ulsan 44919, Korea; bhardwaj.jyoti82@gmail.com

**Keywords:** silica nanoparticles, influenza virus, paper sensor, stencil printing, electrochemical immunosensor, label-free detection, carbon nanotubes

## Abstract

Although many studies concerning the detection of influenza virus have been published, a paper-based, label-free electrochemical immunosensor has never been reported. Here, we present a cost-effective, handmade paper-based immunosensor for label-free electrochemical detection of influenza virus H1N1. This immunosensor was prepared by modifying paper with a spray of hydrophobic silica nanoparticles, and using stencil-printed electrodes. We used a glass vaporizer to spray the hydrophobic silica nanoparticles onto the paper, rendering it super-hydrophobic. The super-hydrophobicity, which is essential for this paper-based biosensor, was achieved via 30–40 spray coatings, corresponding to a 0.39–0.41 mg cm^−2^ coating of nanoparticles on the paper and yielding a water contact angle of 150° ± 1°. Stencil-printed carbon electrodes modified with single-walled carbon nanotubes and chitosan were employed to increase the sensitivity of the sensor, and the antibodies were immobilized via glutaraldehyde cross-linking. Differential pulse voltammetry was used to assess the sensitivity of the sensors at various virus concentrations, ranging from 10 to 10^4^ PFU mL^−1^, and the selectivity was assessed against MS2 bacteriophages and the influenza B viruses. These immunosensors showed good linear behaviors, improved detection times (30 min), and selectivity for the H1N1 virus with a limit of detection of 113 PFU mL^−1^, which is sufficiently sensitive for rapid on-site diagnosis. The simple and inexpensive methodologies developed in this study have great potential to be used for the development of a low-cost and disposable immunosensor for detection of pathogenic microorganisms, especially in developing countries.

## 1. Introduction

Recently, there has been growing demand for low-cost, point-of-care biosensors that require minimal instrumentation, especially in developing countries. Paper-based biosensors are one such class that fulfill these requirements [1]. Paper is a popular substrate material for biosensors because of its excellent characteristics, for example, its mechanical properties such as flexibility, high specific stiffness, and lightness. In addition, its fibrous and porous structure imparts (i) good absorbency (for effective storage and delivery of samples), (ii) air permeability (eliminating air bubbles that are prominent in microfluidics), (iii) a high surface-to-volume ratio (for the increased number of biomolecules to be immobilized), and (iv) capillary action (which obviates the need for pumps to transport fluids) [2].

Most paper-based biosensors can be categorized into optical and electrochemical sensors. Colorimetric detection, such as in a home pregnancy test kit, is widely employed for many paper-based sensors, but only semi-quantification can be achieved with this technique. Therefore, a great deal of research has been directed toward the development of sensitive paper-based biosensors that can provide quantitative information as well, by using surface-enhanced Raman spectroscopy, fluorescence spectroscopy, etc. [3,4].

Electrochemical detection has been used extensively in conjunction with paper-based biosensors because of its low cost, portability, high sensitivity, and high selectivity. There have been many reports concerning electrochemical microfluidic paper-based devices for the detection of various analytes [5,6,7,8,9]. Often, screen printing and inkjet printing have been used to deposit the electrodes onto the paper surface [2]. Recently, Godino et al. [10] presented a cost-effective and facile method for depositing electrodes using a homemade self-adhesive vinyl stencil.

In this study, we present a low-cost paper-based immunosensor for the label-free electrochemical detection of influenza virus H1N1 using paper modified with hydrophobic silica nanoparticles (NPs) and stencil-printed electrodes. Although there have been many reports concerning the detection of influenza viruses [11,12,13,14,15,16,17], paper substrates have infrequently been used. Moreover, most paper-based devices for the detection of infectious diseases have adopted colorimetric detection methods, which are either qualitative or semi-quantitative [18,19,20]. A paper-based electrochemical immunosensor for the detection of the influenza virus has not yet been reported (Table S1).

In the present study, sprayed hydrophobic silica NPs [21] were used to impart super-hydrophobicity to a paper substrate. Three electrodes, used for electrochemical measurements, were left unsprayed. Homemade stencils were used to make the electrodes on the substrate by hand. This simplified the fabrication processes and improved its cost-effectiveness compared to other fabrication methods such as silicon-based microfabrication techniques, and this relatively simple and inexpensive process is especially beneficial for biosensor preparation in developing countries. Single-walled carbon nanotubes (SWCNTs) and chitosan (CH) were employed to modify the working electrode because CNTs provide an ultra-sensitive sensing environment and possess good mechanical and electrical properties [22,23,24,25]. Further, CH has excellent biocompatibility, adhesion, and high water permeability [22]. The details of the overall fabrication process and the label-free electrochemical detection (sensitivity, selectivity, and stability) of influenza H1N1 virus are presented below.

## 2. Materials and Methods

### 2.1. Materials

CNTs (90% SWCNTs, diameter: 1–2 nm, length: 5–30 μm, COOH content: approximately 2.75 wt %) were purchased from MK Impex Corp., Canada. CH (C1098), dimethylformamide (DMF) (98%, D1021), and phosphate-buffered saline (PBS) (10×, pH 7.4) were purchased from Biosesang Inc., Korea. Conductive carbon paste (DC-21, sheet resistance: 20–25 Ω/square at 20 μm thickness) and Ag/AgCl ink were obtained from Dozen Tech., South Korea, and BAS Inc., Tokyo, Japan, respectively. Polydimethylsiloxane (PDMS) modified hydrophobic silica NPs (diameter: 14 nm) were obtained from Plasma Chem. GmbH, Berlin. Whatman chromatography paper 4# (1004-185) was obtained from General Electric Healthcare Worldwide, South Korea. Bovine serum albumin (BSA) (A2153), glutaraldehyde (GA) (G765), and acetate buffer (31048) were obtained from Sigma-Aldrich, USA. Goat anti-influenza A antibody (Ab) (AB1074) was purchased from EMD Millipore. Influenza virus H1N1 (KBPV-VR-33) and influenza virus B (KBPV-VR-34) were procured from the bank of pathogenic viruses, South Korea. Bacteriophage MS2 (ATCC^®^ 15597-B1™, 1 × 10^9^ PFU mL^−1^) was procured from Koram Biogen Corp., South Korea. Deionized water (dH_2_O) (resistance: ~18.2 MΩ) from the Millipore Milli-Q water purification system was utilized for the preparation of the required aqueous solutions (molecular biology grade) and for the dilution of PBS. All solutions and glassware were autoclaved prior to use.

### 2.2. Hydrophobization of Paper

**Scheme 1:**
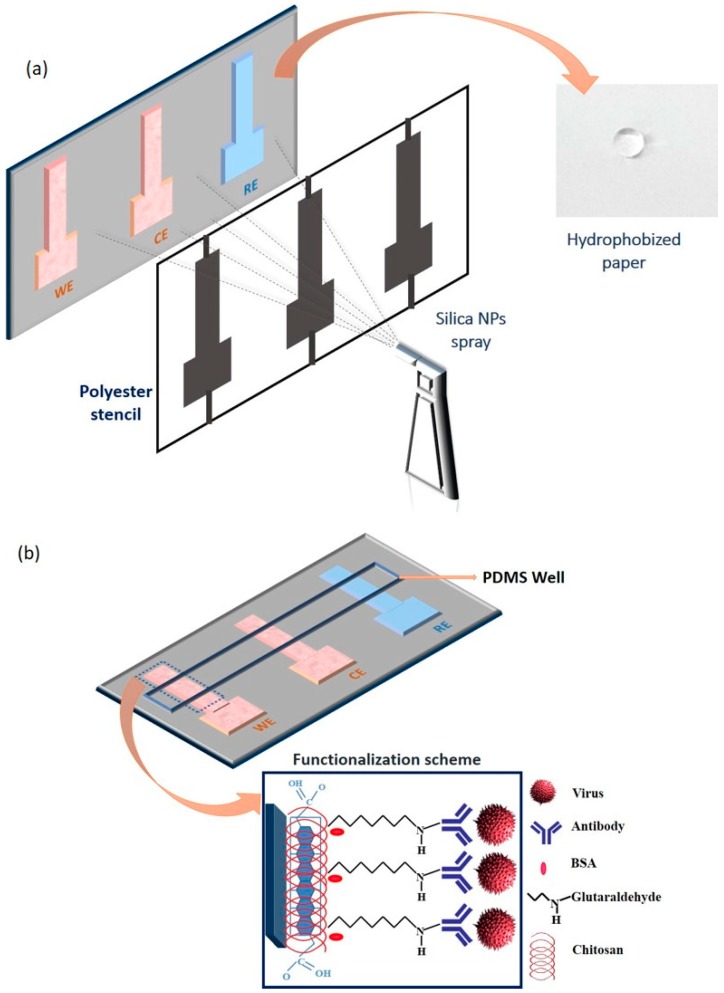
Scheme illustration showing (**a**) the hydrophobization of paper using a glass vaporizer and a polyester stencil, which prevented the silica NPs sticking onto the places for the three electrodes (Note: the distance between the paper and the stencil is exaggerated here for illustration purpose.) and (**b**) the paper-based immunosensor with a PDMS well containing the electrolyte, and the functionalization scheme of the working electrode.

PDMS-modified silica NPs were manually sprayed onto Whatman paper to impart hydrophobicity. A suspension of silica NPs was prepared in ethanol (99.9 %, EMSURE) by adding 0.4 g of NPs into 20 mL of ethanol, followed by stirring for 30 min and sonication for 20 min. The suspension was then sprayed onto selected areas of the paper, excluding the electrode areas, with a glass vaporizer (1180-22, TLC Sprayer, LukeGL^®^, Shinmyung Science, Inc.) and a polyester stencil. This stencil was cut out with a laser cutting machine (VersaLaser). After spraying, the samples were dried in an oven at 50 °C for 2 h (Scheme 1a).

The contact angles (CA) of water droplets on the hydrophobized paper were measured with a contact angle meter (Phoenix 300) to determine the degree of hydrophobicity. The number of silica NPs generated from the spray coatings was also calculated by measuring the weight of the sprayed paper. Eight paper samples were taken, each measuring 3 cm × 3 cm, and the weight of each sample was recorded with a balance (AS 60/220.R2, RADWAG, Poland). These samples were weighed again after the NPs had been sprayed, and the number of sprays was varied from 5 to 40 to estimate the amount of deposited NPs per cm^2^.

### 2.3. Fabrication of the Electrodes onto Paper via Stencil Printing

A three-electrode system containing the working (WE), counter (CE), and reference electrodes (RE) was used for the electrochemical immunoassay. The WE and CE were fabricated with conductive carbon paste, and the RE was made by using Ag/AgCl ink. A low-cost, self-adhesive polyester stencil (opposite in shape to that mentioned earlier for hydrophobization) was utilized for the deposition of the electrodes onto the hydrophilic part of the paper. The polyester stencil (thickness: 100 μm) was placed on the paper to spread the carbon paste uniformly with a squeegee, and the samples were air-dried for 10 min before the stencil was peeled off. The two electrodes were then cured at 70 °C in an oven for 30 min. The entire procedure was repeated to adjust the thickness of the electrodes to 0.1 mm (±0.01 mm), and the electrodes and electrical contact pads measured 1 mm × 7 mm and 3 mm × 3 mm, respectively. Ag/AgCl ink was then applied and dried at room temperature for 30 min to form the RE. After the fabrication of the electrodes, the WE was modified with SWCNTs. The SWCNT suspension was prepared by sonicating the SWCNTs in DMF (concentration: 10 μg mL^−1^) in a water bath for 90 min, followed by centrifugation at 5000 rpm for 1 h, and the supernatant was collected. Then, 5 μL of this suspension was drop-cast onto the WE and dried at room temperature for 12 h. This electrode was then rinsed with dH_2_O and dried with a stream of nitrogen gas.

### 2.4. Bio-Tailoring of the Working Electrode

A CH solution was prepared by dissolving CH (10 mg) in 10 mL of acetate buffer (0.05 M, pH 4.2) and was kept for 4 days at 25 °C to obtain a clear solution. Then, 5 μL of the CH solution was drop-cast onto the CNT/C electrode and dried at room temperature for 2 h. The electrode was then rinsed with dH_2_O and dried with a stream of nitrogen gas. The CH-CNT/C was incubated with 5 μL of 2.5% GA (used as a cross-linking agent) at room temperature for 30 min followed by rinsing with dH_2_O and drying with N_2_ stream. The CH-CNT/C surface was further modified with 5 μL of monoclonal antibodies (10 μg mL^−1^) specific to influenza viruses H1N1 at 37 °C for 2 h. After the electrode had been washed with PBS (1×, pH 7.4), it was incubated in BSA (1 mg mL^−1^) for 30 min to prevent nonspecific binding, and the electrode was then washed with PBS (1×, pH 7.4) again. The Ab-CH-CNT/C was finally incubated in 5 μL of various virus concentrations ranging from 10 to 10^4^ PFU mL^−1^ in PBS (1×, pH 7.4) for 30 min followed by rinsing with PBS (1×, pH 7.4) and distilled water (dH_2_O), sequentially. The samples were then air-dried (Scheme 1b). For the sensor selectivity studies, the electrodes were incubated with MS2 bacteriophages (10^4^ PFU mL^−1^) and influenza B virus (10^4^ PFU mL^−1^) in the same way as for the H1N1 viruses.

### 2.5. Structural and Morphological Characterizations

After each functionalization step, field-emission scanning electron microscopy (FE-SEM) and atomic force microscopy (AFM) were used to characterize the surface morphology of the bio-electrodes (WE). FE-SEM images were obtained using an S-4800 (Hitachi, Tokyo, Japan). A thin platinum layer was deposited at 20 mA for 60 s on the paper substrates by sputtering prior to taking SEM measurements. AFM images were obtained using a Veeco Dimension 3100 (Bruker corp., Billerica, MA, USA). Fourier transform infrared spectroscopy (FTIR) measurements (Varian 4100, Agilent, Santa Clara, CA, USA) were used for structural characterization of the samples.

### 2.6. Electrochemical Measurements

All electrochemical studies were carried out using a potentiostat/galvanostat (Autolab PGSTAT204, Metrohm, Utrecht, The Netherlands) under stationary conditions by using 200 μL of 10 mM PBS (1×, pH 7.4) containing 2.5 mM [Fe(CN)_6_]^−3/−4^ and 100 mM NaCl as the electrolyte. In all the electrochemical experiments, a PDMS well was used to confine the electrolyte on the sensor (Scheme 1b). To check the stability of the device, we prepared the sensors up until the Ab-BSA stage and these were then stored at 4 °C. The electrochemical readings were taken every 3 days for 15 days.

## 3. Results and Discussion

The results of the current study, including (i) characterization (SEM and CA) of the hydrophobized paper, (ii) characterization (SEM, AFM, and FTIR studies) of the bio-electrodes, (iii) electrochemical characterization (cyclic voltammetry (CV), DPV, and electrochemical impedance spectroscopy (EIS) measurements), and (iv) sensitivity, selectivity, and stability studies of the immunosensor for detection of H1N1 viruses are discussed in the following sections.

### 3.1. Superhydrophobicity of the Paper Substrate

**Figure 1:**
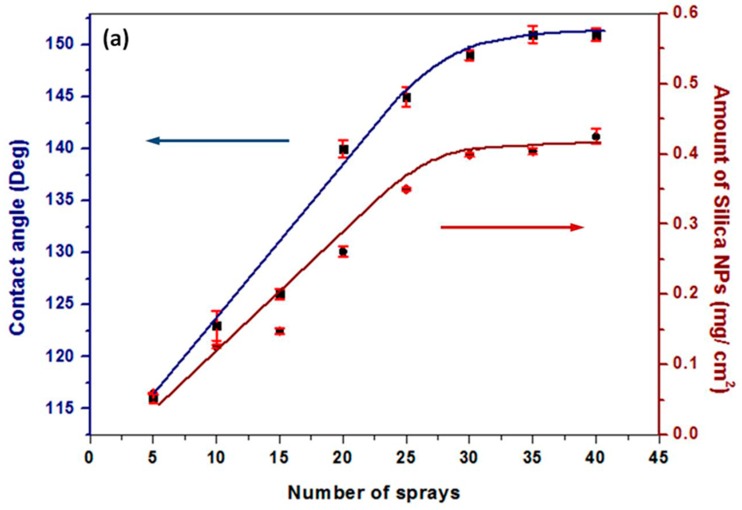

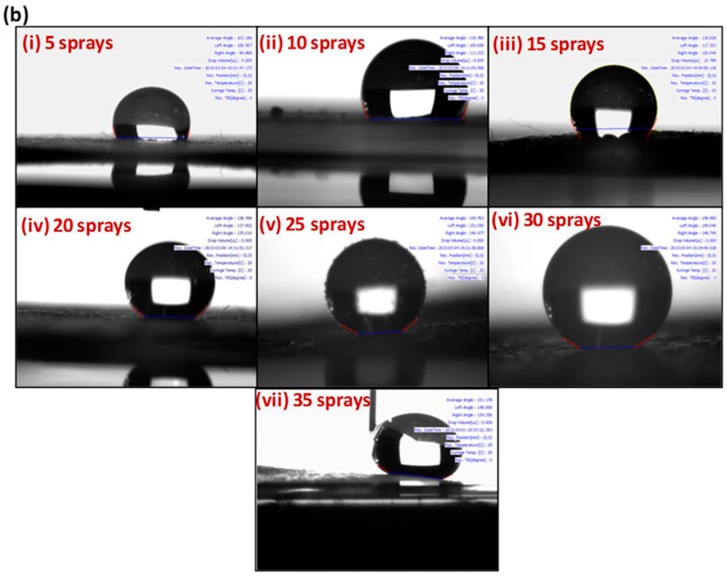
(**a**) Plot showing the variation of the water contact angle and the amount of silica NPs deposited on the paper versus the number of silica NP sprayings. (**b**) Contact angle images for samples prepared with 5 to 35 sprays.

Figure 1a shows the effect of the number of NP sprays on the water contact angle. The contact angle was observed to increase (from 115° to 150°) with increasing spray number (from 5 to 40). This can also be seen from the optical images of water droplets on the paper and the respective contact angles (Figure 1b). The contact angle appeared to stabilize (150° ± 1°) between 30–40 sprays, where the droplets bounced and rolled off the paper surface, demonstrating the super-hydrophobicity of the paper. The amount of silica NPs corresponded to 0.39–0.41 mg cm^−2^ after 30–40 spray coatings, which was determined by weight measurements.

We also tested whether this degree of hydrophobicity was sufficient during the functionalization and electrochemical assay steps (Figure S1). We found that, when the antibodies were immobilized on the CH-CNT/C electrodes by incubation for 2 h followed by washing, the device remained undamaged (Figure S1a). For the electrochemical studies, the paper sensor was able to confine the electrolyte in the PDMS well for a period of 5 min (the entire electrochemical analysis usually took less than 5 min), although this varied with the hydrophobicity of the paper (Figure S1b). When 15 or 25 sprays were used, the sensing device appeared to be damaged by wetting (Figure S1c). However, when 35 sprays were used, sufficient hydrophobicity was obtained. Hence, 35 sprays were used for the functionalization and subsequent electrochemical assay in this study.

**Figure 2:**
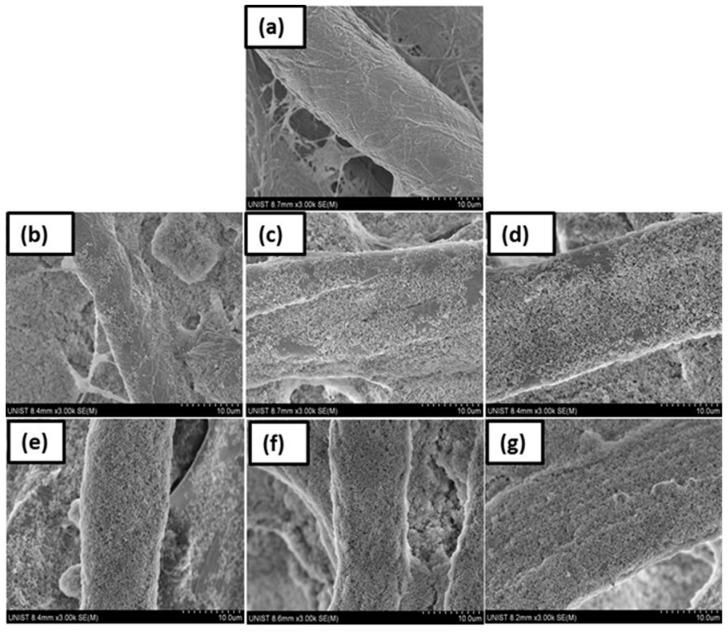
SEM images of bare paper (**a**) and silica NPs on paper after different numbers of sprays, from 5 to 30, at higher magnification, showing single paper fibers (**b**–**g**).

SEM studies were also carried out to study a change in the surface morphology with the number of silica-NP sprays applied onto paper. Figure 2a shows an SEM image of a bare paper surface in which the individual paper fibers can be clearly seen. In Figure 2b–g, silica NPs were observed to cover the paper fibers. Further, the extent of coverage of the NPs increased with the number of sprays, and the entire surface of the paper was covered uniformly after approximately 30 sprays, where paper exhibited maximum hydrophobicity (CA, 150° ± 1°), or super-hydrophobicity. 

The surface hydrophobicity depends on the surface energy and the surface roughness. Ogihara et al. [21] found that when a silica NP suspension in ethanol was spray-coated onto paper, a hierarchical rough surface was formed. That is, nanometer-sized roughness was added on top of a microscale rough surface, and this was known to be responsible for the super-hydrophobicity. This type of structure was also apparent from the SEM images taken in the present study (Figure 2b–g). In fact, the SEM images at low magnification revealed the fibrous structure, or microscale roughness of the paper (Supplementary data, Figure S2a–g), along with the nanoscale roughness induced by the NPs. However, the hierarchical roughness was not observed when suspension of higher alcohols, such as 1-propanol and 1-butanol, were used [21]. Hence, we prepared the silica NP suspensions in ethanol.

### 3.2. Morphological and Structural Characterizations of the Bio-Electrodes

**Figure 3:**
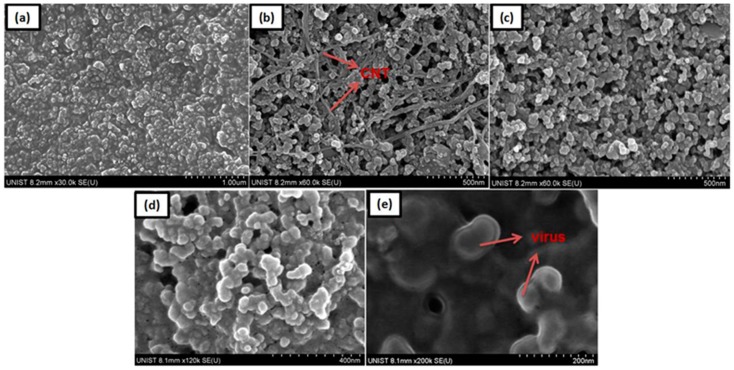
SEM images of (**a**) bare carbon, (**b**) CNT/C, (**c**) CH-CNT/C, (**d**) Ab-CH-CNT/C, and (**e**) virus-Ab-CH-CNT/C.

Figure 3 shows SEM images for (a) bare carbon on paper, (b) CNT/C, (c) CH-CNT/C, (d) Ab-CH-CNT/C, and (e) virus-Ab-CH-CNT/C. The SWCNTs can be clearly seen (Figure 3b) and were mostly assembled by physical adsorption on the rough carbon surface. Figure 3c shows CH covering up the SWCNT network. CH was deposited on the CNT/C electrode by covalent binding, and creates a favorable environment for further immobilizations of antibody and virus. The globular and much smoother surface was evident after the antibodies were immobilized by covalent binding using GA as a cross-linking agent (Figure 3d). A bare carbon surface showed the highest average roughness (*R_a_*: 42.2 nm), and the average roughness decreased with each subsequent immobilization. The *R_a_* values measured from AFM were found to be 37.7, 34.1, and 18 nm after SWCNT, CH, and Ab depositions, respectively (Figure S3a–c). Figure 3e shows an SEM image of the influenza viruses (diameter: approximately 96 nm) captured by the antibody. The average size of a single influenza virus ranged from 80 to 120 nm [26]. Therefore, we concluded that the functionalizations were successful, and this was further verified by FTIR studies (Figure S3d).

### 3.3. Electrochemical Characterizations of the Bio-Electrodes

The incubation time for the drop-cast SWCNTs on the carbon surfaces of the bio-electrodes plays a crucial role in the proper deposition of the SWCNTs [27]. Therefore, CV and EIS studies were conducted to find the optimal incubation time for the drop-cast SWCNTs. The SWCNTs were first drop-cast on the carbon electrodes and incubated at room temperature for 2, 4, 6, and 12 h. The electrochemical responses of these samples were then recorded, and these are presented in Figure S4. As the incubation time increased from 2 to 12 h, the current increased, and the charge transfer resistance (R_ct_) decreased (Figure S4a,b). The changes in the current and R_ct_ were very large up to 4 h, then gradually became smaller until finally reaching a saturated value for the incubation time of 12 h (Figure S4a,b(iv)). Hence, in this study, a 12 h incubation time was used for the drop-cast SWCNTs.

**Figure 4:**
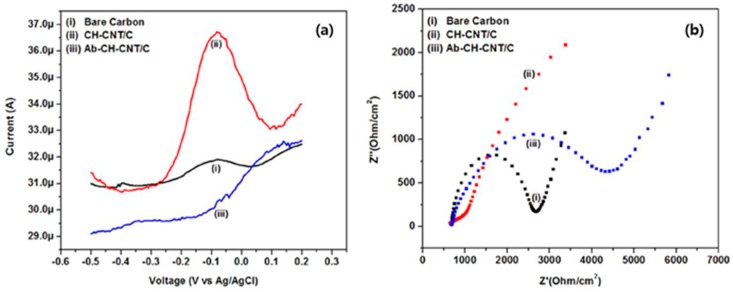
(**a**) Differential pulse voltammograms and (**b**) impedance spectra for characterization of (i) bare carbon, (ii) CH-CNT/C, and (iii) Ab-CH-CNT/C in 2.5mM [Fe(CN)_6_]^−3/−4^.

SWCNT, CH, and Ab functionalized electrodes were also characterized by DPV and EIS measurements to confirm their deposition. Figure 4a,b shows the DPV and EIS spectra for (i) bare carbon, (ii) CH-CNT/C, and (iii) Ab-CH-CNT/C bio-electrodes. From the DPV curves (Figure 4a), we observe that the peak current increased from 31.91 μA (bare carbon) to 36.73 μA after the deposition of SWCNTs and CH onto the carbon electrode. Similarly, from the EIS spectra (Figure 4b), R_ct_ decreased from 2.10 kΩ (bare carbon) to 0.16 kΩ (CH-CNT/C). This increase is expected because CNTs have excellent electrical properties, allowing for efficient electron transfer during the redox reaction [28,29]. With the application of antibodies onto the CH-CNT/C, the peak current (30.52 μA) decreased, and R_ct_ (5.70 kΩ) increased further. This may be due to the insulating nature of the antibodies blocking the [Fe(CN)_6_]^3−/4−^ ions from reaching the electrode surface, thereby preventing electron transfer. This result demonstrates the successful immobilization of the antibodies on the CH-CNT/C surface.

### 3.4. Sensitivity and Selectivity Studies

**Figure 5:**
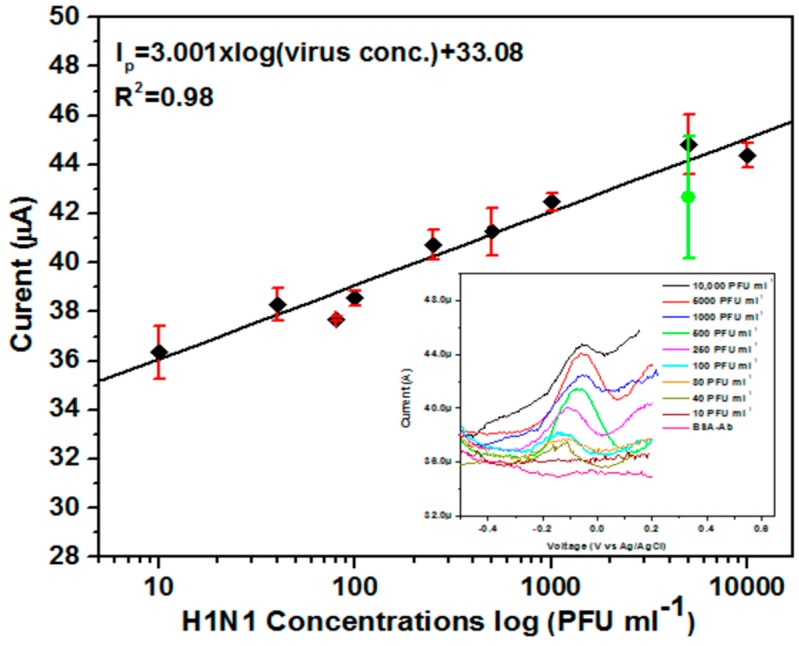
Calibration curves obtained for varying concentrations of H1N1 virus from 10 to 10^4^ PFU mL^−1^ in PBS and in saliva (5000 PFU mL^−1^, green symbols). The error bars show the standard deviations of the measurements.

The behavior of the immunosensors fabricated in this study was assessed by performing DPV. To evaluate the limit of detection (LOD) of these sensors, we diluted the H1N1 viral solutions in PBS (1×, pH 7.4), resulting in virus concentrations ranging from 10 PFU mL^−1^ to 10^4^ PFU mL^−1^. Then, 5 μL of each viral solution was applied to the Ab-CH-CNT/C electrodes. The peak current exhibited a good linear relationship with the logarithmic value of virus concentration (Figure 5). The slope and intercept were calculated to be 3.001 and 33.08 with standard deviations of 0.406 and 1.074, respectively. Moreover, the peak current was observed to increase with increasing viral concentration. Similar observations were made in several label-free electrochemical studies [16,30,31,32,33]. The increase in the peak current with viral concentrations might be due to the reaction between antigen and antibody, which might create favorable orientations for easy electron transfer to the electrode surface; however, no clear mechanism has been established yet [30,31,32,33].

Further, the LOD was calculated from the linear calibration equation (Figure 5) obtained for the immunosensors using the signal to noise ratio (S/N) method by employing a factor of 3 and found to be 113 PFU mL^−1^. In addition, the detection or incubation time of the immunosensor was 30 min. The LOD and detection time of the present paper-based electrochemical immunosensor may not be comparable to those of the immunosensors using other substrates. In fact, Kiilerich-Pedersen et al. [12] made an aptamer-based sensor using a polymer system for detecting influenza virus electrochemically, and the LOD of this sensor was as low as 10 PFU mL^−1^. Singh et al. [15,16] presented a carbon nanotube-based electrical immunosensor using a silicon substrate and a graphene-based electrochemical immunosensor integrated with a microfluidic platform for the detection of influenza virus H1N1, with the LODs being 1 PFU mL^−1^ and 0.5 PFU mL^−1^, respectively. However, considering that the range of influenza viral particles found in the infected swine nasal samples is approximately 10^3^–10^5^ PFU mL^−1^ [34], the present paper sensor is sufficiently sensitive, and has the potential for rapid on-site diagnosis.

To check the performance of the immunosensor in a more realistic environment, the saliva obtained from a healthy person was diluted with PBS, and the sample was spiked with H1N1 viruses at a concentration of 5000 PFU mL^−1^. This immunosensor was also able to detect the virus in the saliva sample, as indicated by the peak current, which slightly overlapped with that of H1N1 viruses diluted only in PBS at the same concentration (Figure 5, green symbol). However, there was an increase in the noise (larger measurement uncertainty) in the saliva sample. Similar observation of increased noises was also made in the study conducted by Hewa et al. [11], in which nasal wash samples with simple pre-treatment were used to detect influenza virus, and an increase in noise was reported. The DPV measurements for blank PBS, blank saliva, and virus-spiked saliva samples are shown in Supplementary Information (Figure S5). 

**Figure 6:**
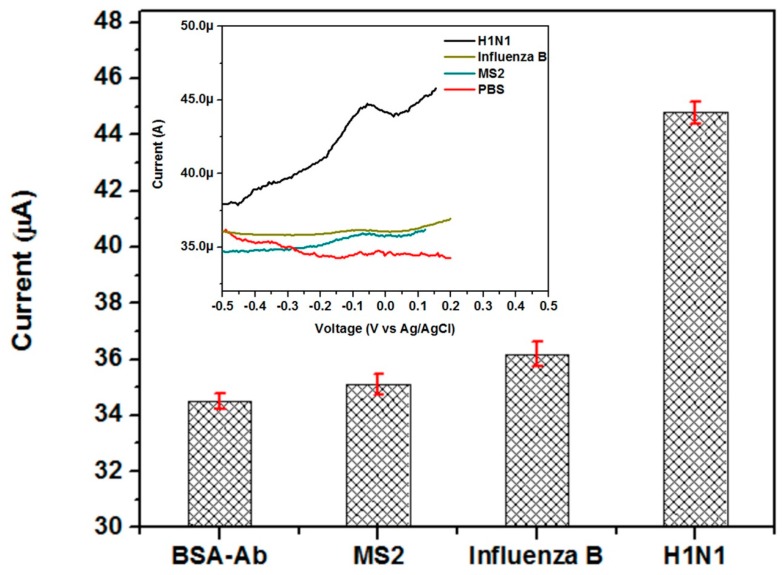
Selectivity test of the sensor for H1N1 virus with respect to the MS2 bacteriophage (10^4^ PFU mL^−1^) and influenza B virus (10^4^ PFU mL^−1^) diluted in PBS (1×, pH 7.4). The error bars show the standard deviations of the measurements.

The selectivity of the present immunosensor was also assessed by carrying out DPV with MS2 bacteriophages (10^4^ PFU mL^−1^) and influenza B virus (10^4^ PFU mL^−1^) diluted in PBS (1×, pH 7.4). The DPV responses (Figure 6) showed almost negligible peak current for MS2 and influenza B viruses, exhibiting almost the same peak currents as the PBS only case. In contrast, they showed a distinguishable peak in the case of H1N1 at the same concentration, i.e., 10^4^ PFU mL^−1^, demonstrating the excellent specificity for H1N1 viruses. These sensors also showed good stability for 15 days when the sensing performance was evaluated every 3 days after storing the Ab-BSA immobilized sensors at 4 °C. The relative standard deviation, which can be defined as the standard deviation of the peak currents divided by the mean value of the peak currents, was estimated to be 2.6% after 6 days and 4.4% after 15 days. Furthermore, the dissociation constant (K_D_) was calculated using DPV based on the Langmuir isotherm model [35], and was measured to be 154 ± 36 PFU mL^−1^ (Figure S6).

## 4. Conclusions

We have presented a simple-to-prepare, cost-effective, and label-free electrochemical immunosensor consisting of silica nanoparticles modified paper and stencil-printed electrodes. This is the first paper-based electrochemical immunosensor for the detection of an influenza virus in PBS (artificial environment) and in saliva (realistic environment). In addition, the present preparation method is facile and requires few resources, requiring only spraying, via a glass vaporizer, of silica NPs to impart super-hydrophobicity to paper. Spraying the paper 30–40 times yielded the maximum hydrophobicity, which was shown to be sufficient for functionalization and electrochemical assay. In addition to reducing the total cost (<1 USD for main materials), these immunosensors showed sufficiently high sensitivity (LOD: 113 PFU mL^−1^), stability (relative standard deviation: 4.4% after 15 days), selectivity (against MS2 and influenza B viruses), and high linearity (*R*^2^ = 0.98) in the concentration range of 10 to 10^4^ PFU mL^−1^ with a detection time of 30 min. This immunosensor was hand-made using simple tools, and it shows great promise for the development of disposable point-of-care devices for use in developing countries.

## Supplementary Materials

The following are available online at http://www.mdpi.com/1424-8220/17/11/2597/s1. Figure S1: (a) Immobilization and washing steps (b) Images of the paper substrates before and after electrochemical studies conducted for 5 min for varying number of sprays (15, 25, and 35 sprays) (c) Damaged sensors: showing damage due to short circuit at the working electrode (left) and tearing while handling (right) desired hydrophobicity level is not reached, Figure S2: SEM images of bare paper (a), silica NPs on paper with varying number of sprays from 5 to 30 at lower magnification (b–g) and at higher magnification for 30 sprays (h,i), Figure S3: AFM images of (a) CNT/C (b) CH-CNT/C (c) Ab-CH-CNT/C, and (d) FTIR for (i) CNT/C (ii) CH-CNT/C (iii) Ab-CH-CNT/C bio-electrodes, Figure S4: (a) Cyclic voltammograms and (b) impedance spectra for standardization of incubation time for the drop-cast SWCNTs on the carbon surfaces of the bio-electrodes, Figure S5: Differential pulse voltammograms for blank saliva, blank PBS and virus (5000 PFU mL-1) containing saliva samples, Figure S6: Binding kinetics of the adsorption of influenza viruses to the antibody modified electrode, Table S1: Comparison of previous works on paper based sensors for detection of viruses with present work.
