# Supplementary material for: Cost-Effective and Handmade Paper-Based Immunosensing Device for Electrochemical Detection of Influenza Virus
Source: Sensors (Basel). 2017 Nov 11;17(11):2597. doi: 10.3390/s17112597 (PMC5713655; doi:10.3390/s17112597)
Supplement: Supplementary file 1 [file sensors-17-02597-s001.pdf]

Supplementary Information

Cost-effective and handmade paper-based immunosensing device for electrochemical detection of influenza virus

Sivaranjani Devarakonda[a,^], Renu Singh[a,^], Jyoti Bhardwaj[b], Jaesung Jang [a,b,†]

[a] Department of Mechanical Engineering, School of Mechanical, Aerospace and Nuclear Engineering, Ulsan National Institute of Science and Technology (UNIST), Ulsan 44919, Republic of Korea

[b]Department of Biomedical Engineering, UNIST, Ulsan 44919, Republic of Korea

^Sivaranjani Devarakonda and Renu Singh contributed equally to this work.

†Correspondence should be addressed to jjang@unist.ac.kr; Tel: +82-52-217-2323; Fax: +82-52-217-2449

1. Images showing the effect of hydrophobicity for biosensing applications

(a)

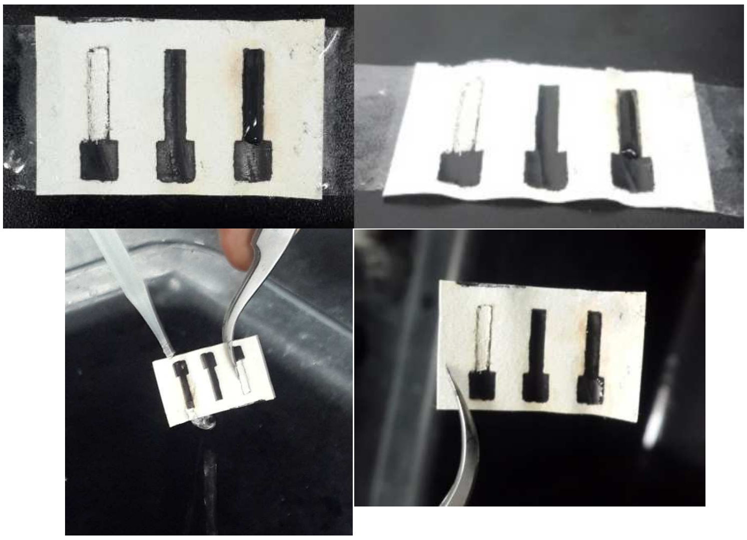

(b)

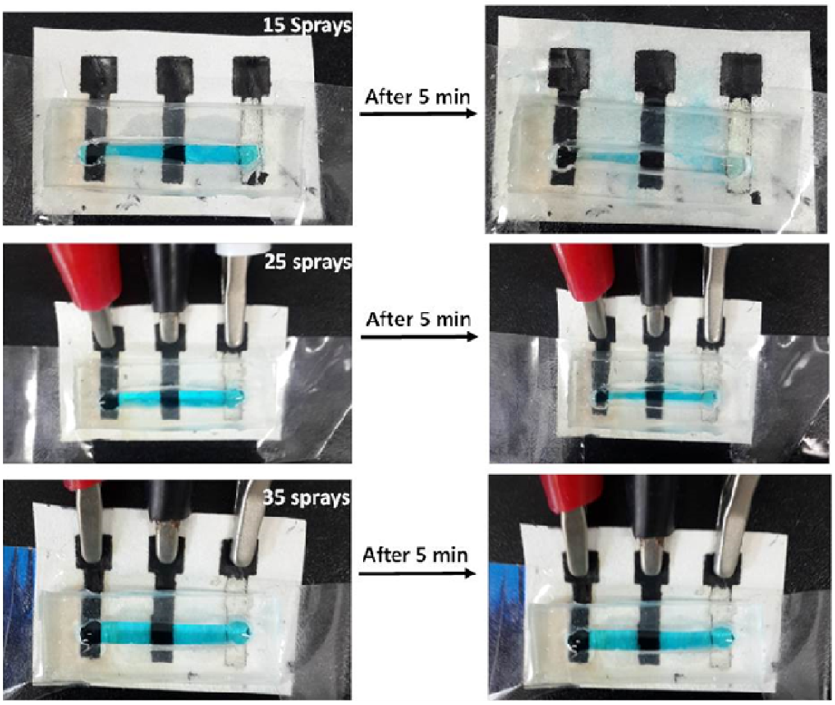

(c)

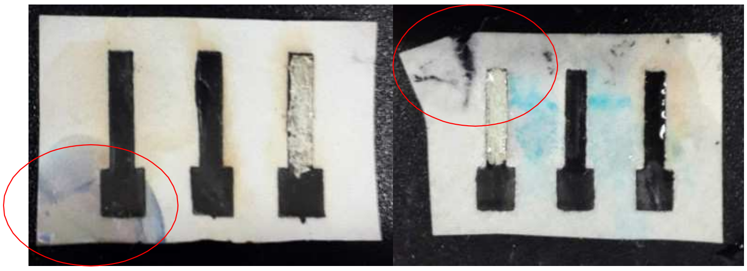

Figure S1: (a) Immobilization and washing steps (b) Images of the paper substrates before and after electrochemical studies conducted for 5 min for varying number of sprays (15, 25, and 35 sprays) (c) Damaged sensors: showing damage due to short circuit at the working electrode (left) and tearing while handling (right) desired hydrophobicity level is not reached.

2. SEM imaging

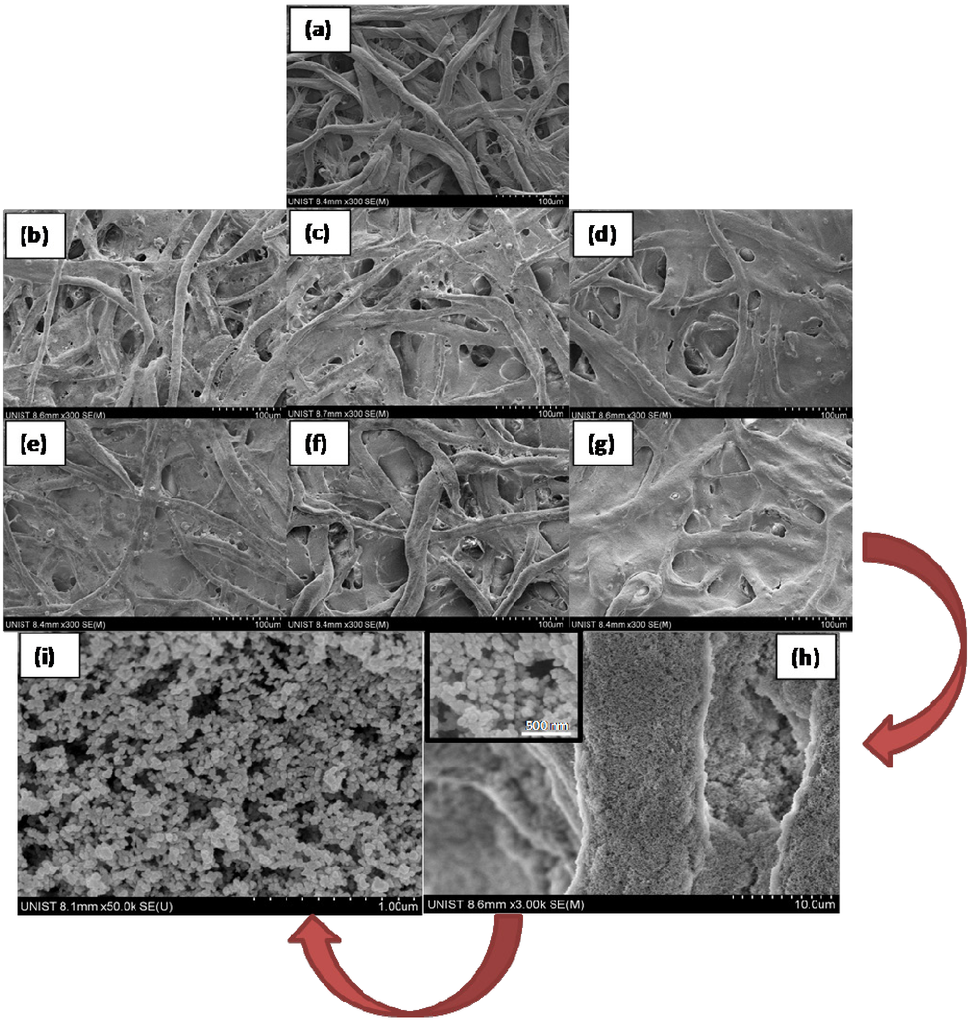

Figure S2: SEM images of bare paper (a), silica NPs on paper with varying number of sprays from 5 to 30 at lower magnification ((b) to ((g)) and at higher magnification for 30 sprays ((h) and (i)).

3. AFM images and FTIR spectroscopic analysis

Figure S3 shows AFM images and FTIR spectra for (i) CNT/C, (ii) CH-CNT/C, (iii) Ab-CH-CNT/C bio-electrodes. In spectrum (i), peaks at 1570 cm^{-1} and 1635 cm^{-1} are associated with the stretching of carbon nanotubes backbone. Peaks at 3022 cm^{-1}, and 1404 cm^{-1} show the O-H stretching and O–H bending deformation in carboxylic acid groups respectively, whilst increased strength of the signal at 1226 cm^{-1} may be associated with C–O stretching in the same functionalities. Spectrum (ii) shows the peaks of CH-CNT/C electrode at 3125 to 3370 cm^{-1} (O–H and N–H stretching); 2923 and 2854 cm^{-1} (C–H stretching of CH_2 groups); 1631 cm^{-1} (C=O stretching of carbonyl group); 1556 cm^{-1} (C=C stretching of CNTs). Spectrum (iii) shows the antibody immobilization onto the CH-CNT/C electrode and the bands at 1665 and 1545 cm^{-1} exhibited due to the primary amide and secondary amide linkages. The band at 3282 cm^{-1} is associated with the combination of the amide and amine N-N frequencies, and corresponds to N-H stretching vibrations.

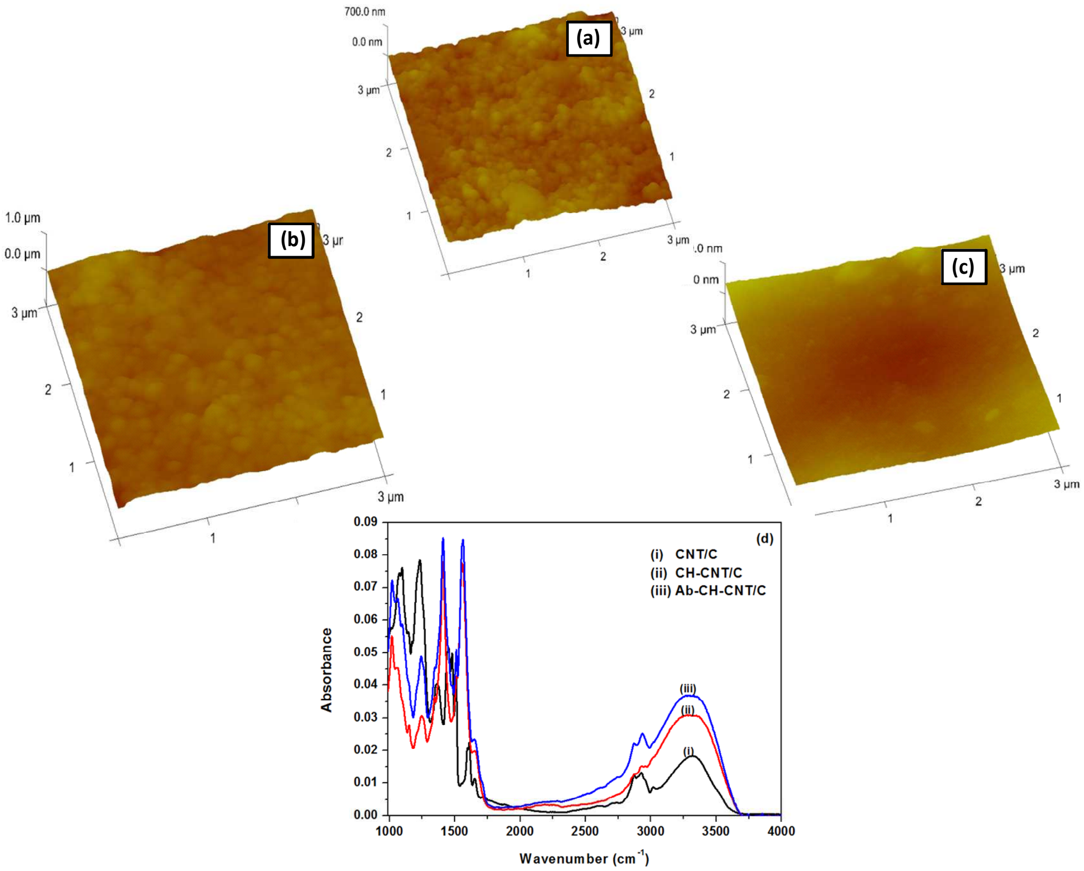

Figure S3: AFM images of (a) CNT/C (b) CH-CNT/C (c) Ab-CH-CNT/C, and (d) FTIR for (i) CNT/C (ii) CH-CNT/C (iii) Ab-CH-CNT/C bio-electrodes.

4. Standardization of incubation time

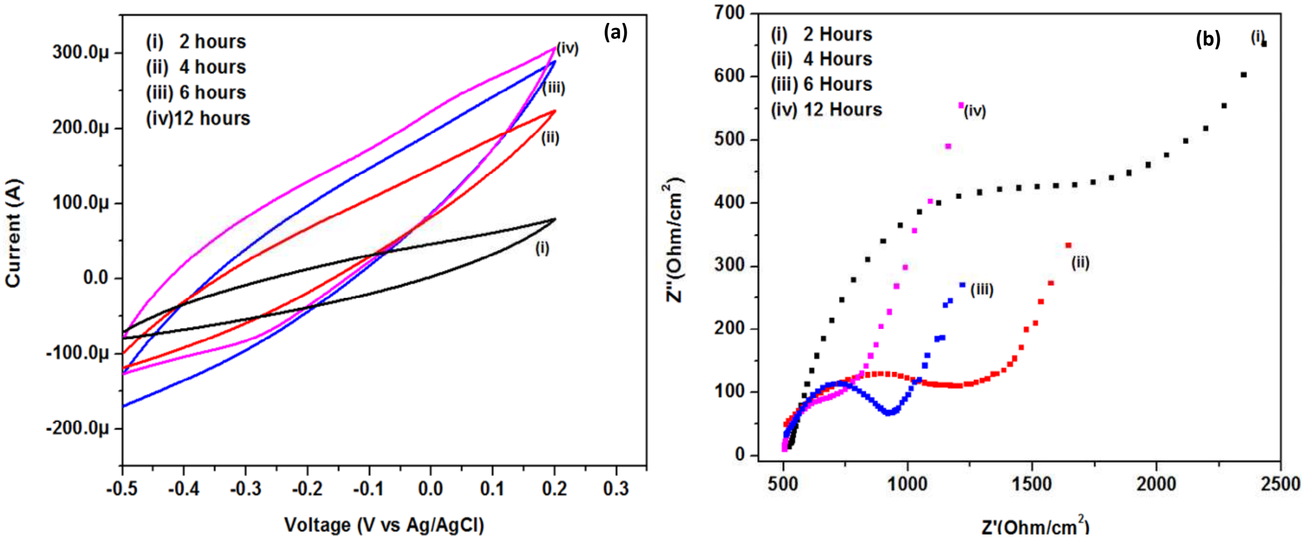

Figure S4: (a) Cyclic voltammograms and (b) impedance spectra for standardization of incubation time for the drop-cast SWCNTs on the carbon surfaces of the bio-electrodes.

5. Electrochemical measurements for detection of virus in saliva

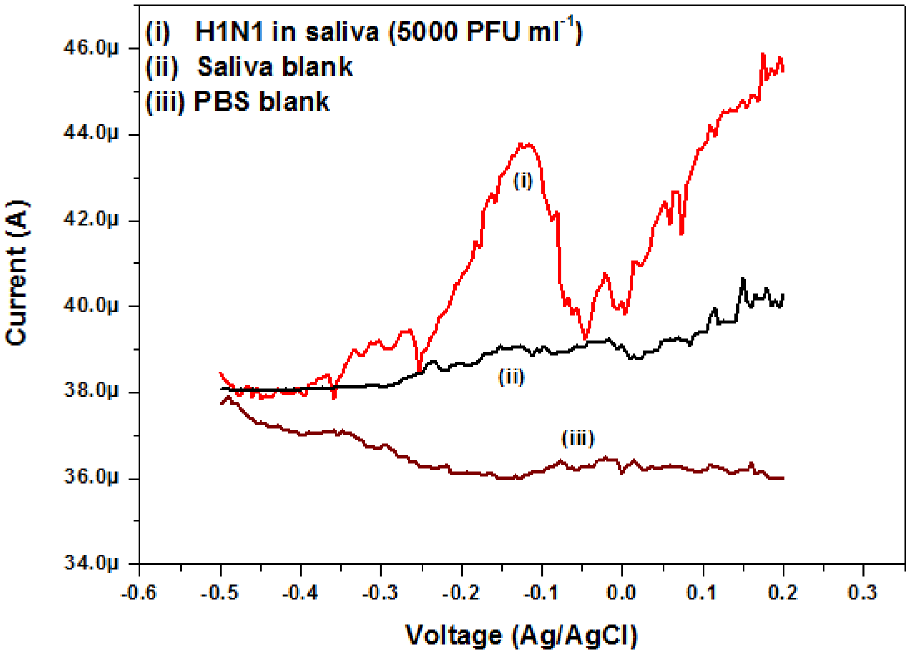

Figure S5: Differential pulse voltammograms for blank saliva, blank PBS and virus (5000 PFU mL^{-1}) containing saliva samples.

6. Determination of the dissociation constant (K_D)

The binding kinetics on the adsorption of influenza viruses to the antibody modified electrode was measured by calculating the dissociation constant (K_D) using DPV. This was based on the Langmuir isotherm model, and this was done by applying the non-linear equation shown below, where I_S was the normalized current, S is the concentration of virus (PFU/mL), and I_{max} is the maximum current (Moreira et al. 2013; Gao et al. 2016). This calibration curve followed the typical behavior of antibody-antigen interaction (Moreira et al. 2013; Gao et al. 2016). Fitting the data to the hyperbolic function (Langmuir), we have got values of K_D=154 ± 36 PFU mL^{-1} and I_{max}=9.4 ± 0.5 µA.

$$I_S = \frac{I_{max}}{1 + K_D/[S]}$$

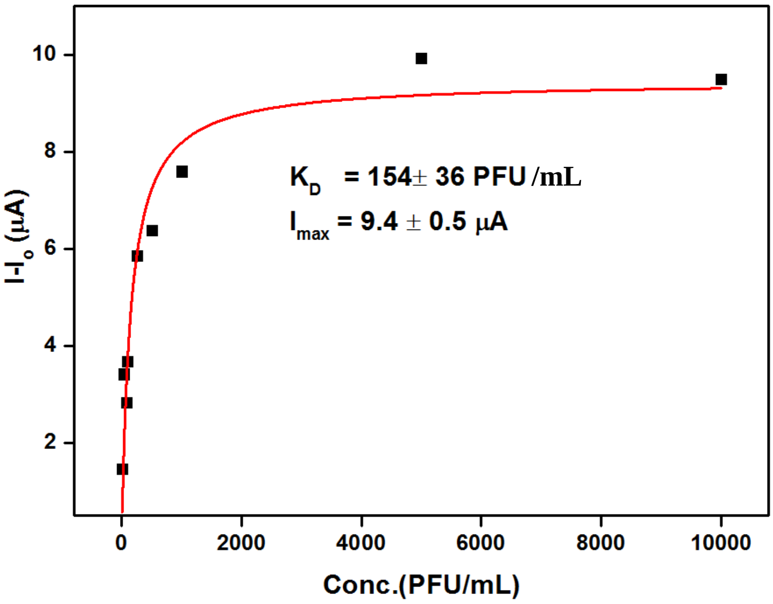

Figure S6: Binding kinetics of the adsorption of influenza viruses to the antibody modified electrode

Table S1: Comparison of previous works on paper based sensors for detection of viruses with present work

Method of paper hydrophobization	Detection method	Analyte detected	Media	Limit of detection	References
Wax printing	Colorimetric (Sandwich immunoassay)	Influenza (H1N1 & H3N2)	PBS	2.7×10^3 PFU/assay* for H1 and 2.7×10^4 PFU/assay* for H3	Lei et al., 2015
Photolithography (SU-8 photoresist embedded paper)	Colorimetric (Indirect ELISA)	HIV-1 (gp41 antigen)	Human Serum	4 fmol/zone	Cheng et al., 2010
Nitrocellulose Membrane used as substrate (half sandwich assay)	Surface Enhanced Raman Scattering (antigen-antibody binding)	Influenza virus (H1N1, H3N2, H5N1)	PBS	30 ng/mL	Lin et al., 2014
Craft punch patterning on Nitrocellulose	Chemiluminescence and colorimetric immunosensor (Indirect ELISA)	HCV	Human serum	267 amol in Chemiluminescence and 26.7 fmol in colorimetric	Mu et al., 2014
Wax printing	Electrochemical immunosensor (Indirect ELISA)	HIV and HCV	Mouse serum	300 pg/mL for HIV and 750 pg/ml for HCV	Zhao et al., 2015
Wax printing (o-PAD*)	Electrochemical sensor	Hepatitis B	PBS	85pM	Li et al., 2015
**Spraying hydrophobic Silica nano-particles**	**Label-free Electrochemical immunosensor**	**Influenza virus (H1N1)**	**PBS and Saliva**	**592 PFU ml^{-1}**	**Present study**

*o-PAD – Origami based paper analytical device

References

Cheng et al. 2010. Angew. Chem. Int. Ed. 49, 4771–4774.

Gao et al. 2016. Proceedings of the National Academy of Sciences 113, 14633-14638.

Lei et al. 2015. Anal. Chim. Acta 883, 37–44.

Li et al. 2015. Anal. Chem. 87(17), 9009-9015.

Lin et al. 2014. Anal. Chem. 86, 5338−5344.

Moreira et al. 2013. Biosensor and Bioelectronics 45, 237-244.

Zhao et al. 2015. Transducers, Anchorage, Alaska, USA, 1025-1028
